# Anisogamy and sex roles: a commentary

**DOI:** 10.1093/evlett/qrae058

**Published:** 2024-10-23

**Authors:** Judit Mokos, István Scheuring, András Liker, Robert P Freckleton, Tamás Székely

**Affiliations:** Department of Ethology, Eötvös Loránd University, Budapest 1117, Hungary; HUN-REN Centre for Ecological Research, Institute of Evolution, Budapest 1121, Hungary; HUN-REN-PE Evolutionary Ecology Research Group, Department of Limnology, University of Pannonia, Veszprém 8201, Hungary; Ecology and Evolutionary Biology, School of Biosciences, University of Sheffield, Western Bank, Sheffield S10 2TN, United Kingdom; Department of Life Sciences, Milner Centre for Evolution, University of Bath, Bath BA2 7AY, United Kingdom; HUN-REN-DE Reproductive Strategies Research Group, Department of Evolutionary Zoology, University of Debrecen, Debrecen 4032, Hungary

**Keywords:** anisogamy, sexual selection, Bateman’s principle, adult sex ratio

## Abstract

The origin and maintenance of sex differences in reproductive behavior (often labeled sex roles) have remained controversial topics, and recent meta-analyses and theoretical models have helped to elucidate the processes that generate diverse sex roles. We are glad to see that our study (Mokos et al., 2021) generated a healthy debate, and in agreement with recent commentaries (Janicke, 2024; Lehtonen & Parker, 2024) we call for a more comprehensive approach to understanding sex role evolution.

*“In sum, sex roles are variable, even though the intensity of intrasexual selection is usually strongly male biased, as predicted by the Darwin–Bateman paradigm. Moreover, this strong sex bias cannot be explained by stochasticity, environmental variation or anisogamy alone.”* (Kappeler et al., 2023)

The Darwin-Bateman Paradigm (DBP) is emerging as a central (albeit controversial) concept in studies of sexual dimorphism, sexual selection, and sex differences in reproduction, and two recent meta-analyses [(Janicke et al., 2016; Mokos et al., 2021) JEA and MEA, respectively] elicited a forward-looking discussion on the emergence of sexual selection and sex roles [(Janicke, 2024; Lehtonen & Parker, 2024), JAN and L&P, respectively]. The arguments have been summarized by L&P and JAN, and here we wish to comment only on two issues.

First, the main motivation of MEA was to revisit one specific pathway of DBP (Pathway 1 that links anisogamy and sexual selection), because we felt that a binary trait (i.e., biological sex represented by anisogamy that emerges from the males producing smaller gametes than the females) used by JEA as a proxy for anisogamy is unable to predict the immense diversity of sexual selection displayed by males and females in nature. To revisit Pathway 1, MEA used two variables representing anisogamy: (a) gamete size bias that indicates male gamete size relative to female gamete size, and (b) gametic investment bias representing testis mass relative to clutch investment accounting for body size. Using these continuous measures, MEA found no association between the extent of anisogamy and the intensity of sexual selection as indicated by three relative measures of sex difference in pre-copulatory sexual selection used by JEA. The latter results were confirmed by the re-analyses of JAN.

We entirely agree with L&P that the lack of association may be due to a ceiling effect, and we clearly stated this explanation in the third paragraph of the Discussion (MEA). The lack of association is also consistent with theoretical expectations (Lehtonen & Parker, 2024; Lehtonen et al., 2016). However, quantitative tests between the extent of anisogamy and sexual selection intensity are important, since theoretical models provide different predictions in regard to the extent of anisogamy and aspects of sex roles (Iyer et al., 2020; Siljestam & Martinossi-Allibert, 2024). Thus the saturation effect predicted by L&P is one of several theoretically possible associations between anisogamy, sexual selection, and sex roles (Henshaw et al., 2022; Janicke, 2024) and further theoretical and empirical work is needed to judge which theoretical model has “broader relevance” and provide “more realistic” predictions for the tree of life.

The second main point of MEA was that anisogamy alone cannot possibly predict the diversity of sex roles observed within a single species or between different species (Gonzalez-Voyer et al., 2022; Jaeggi et al., 2020; Székely et al., 2024). First, sex roles may change dynamically within the same population and it seems unlikely that gametic traits alone (i.e., gamete sizes and their numbers) would drive the sex role variations. For instance, courtship behavior shows temporal variation in katydids and gobies without an apparent shift in the relative gamete sizes of males and females (Forsgren et al., 2004; Hare & Simmons, 2020). Second, in studies of sex-role reversed species, that is, those that exhibit more courtship and less parenting by the females than by the males, ecological factors (e.g., resource distributions) seem to play a key role in predicting sex role variation. Recent studies also suggest that adult sex ratios (i.e., the proportion of males in the adult population) modulate sex role behavior in a number of organisms (Fresneau et al., 2024; Schacht et al., 2022), although corresponding changes in the extent of anisogamy have not been ruled out by the latter studies.

In conclusion, we welcome the propositions of L&P and JAN and appreciate their intention to reconcile seemingly discordant studies. It is encouraging that the overall direction of the discussion on sexual selection and sex roles—starting with the models by Lehtonen et al. (2016) and Parker (2014), and followed by empirical studies by JEA and MEA—have the potential to move this research field forward. We concur with L&P and JAN that to understand the operation of sexual selection, and the emergence and maintenance of diverse sex roles, we will need more comprehensive theoretical and empirical studies (see also Siljestam & Martinossi-Allibert, 2024). Future studies should also consider the ecological and evolutionary feedback relationships between gametic traits, behavior, life histories, and sex ratios (Liker et al., 2013; Long et al., 2024; Székely et al., 2000). Our take-home message is that to uncover the causes of the immense natural variation in sexual selection and sex roles, the number and sizes of gametes are important but not sufficient components, and we also need to consider the various ecological, behavioral, life-history, and demographic processes—and possibly their co-evolutionary relationships.
